# Unveiling the mechanism of photothermal therapy in acne man-agement: targeting sebaceous gland ferroptosis via umbilical cord mesenchymal stem cell membrane-encapsulated Au-Ag-PDA

**DOI:** 10.3389/fbioe.2024.1426477

**Published:** 2024-06-10

**Authors:** Ronghui Wu, Jing Li, Hao Tian, Dandan Song, Tianqi Zhao, Yangyang Tian, Christos C. Zouboulis, Jinlan Jiang, Mingji Zhu

**Affiliations:** ^1^ Department of Dermatology, China-Japan Union Hospital of Jilin University, Changchun, China; ^2^ Scientific Research Center, China-Japan Union Hospital of Jilin University, Changchun, China; ^3^ Department of Orthopedics, China-Japan Union Hospital of Jilin University, Changchun, China; ^4^ Departments of Dermatology, Venereology, Allergology and Immunology, Staedtisches Klinikum Dessau, Brandenburg Medical School Theodor Fontane and Faculty of Health Sciences Brandenburg, Dessau, Germany

**Keywords:** proteomics, Au-Ag-PDA@MSCM, photothermal therapy, ferroptosis, acne, Acsl4

## Abstract

**Background:**

Branched gold and silver nanoparticles coated with polydopamine (Au-Ag-PDA) demonstrate high photothermal conversion efficiency. Utilizing umbilical cord mesenchymal stem cell membranes (MSCM) as an effective drug delivery system, our preliminary studies investigated the suppression of sebum secretion in sebaceous glands using MSCM-coated Au-Ag-PDA nano-particles (Au-Ag-PDA@MSCM) combined with 808 nm laser irradiation, showing potential for dermatological applications in acne treatment.

**Methods:**

This study employs proteomic analysis, complemented by subsequent techniques such as Western blotting (WB), small interfering RNA (siRNA), and transmission electron microscopy, to further investigate the differential mechanisms by which Au-Ag-PDA and Au-Ag-PDA@MSCM-mediated photothermal therapy (PTT) suppress sebum secretion.

**Results:**

Our proteomic analysis indicated mitochondrial respiratory chain damage in sebaceous gland tissues post-PTT, with further validation revealing ferroptosis in sebaceous cells and tissues. Acyl-CoA Synthetase Long-Chain Family Member 4 (Acsl4) has been identified as a critical target, with Au-Ag-PDA@MSCM demonstrating enhanced ferroptotic effects.

**Conclusion:**

These findings significantly advance our understanding of how PTT mediated by Au-Ag-PDA@MSCM nanoparticles reduces sebum secretion and underscore the pivotal role of MSCM in inducing ferroptosis in sebaceous glands, thus providing a robust theoretical foundation for employing PTT via specific molecular pathways in acne treatment.

## 1 Introduction

Acne vulgaris is a chronic inflammatory skin disease affecting the sebaceous glands within hair follicles and is prevalent among adolescents and young adults. Manifesting as acne, papules, and pustules, this condition predominantly appears on the face, chest, and back (Wei et al., 2021). Given its potential to result in lasting facial scarring or hyperpigmentation, acne often exerts a detrimental psychosocial impact (Firlej et al., 2022). The pathogenesis of acne is complex and not entirely understood. The current consensus attributes acne development to multifaceted factors, including excessive sebum production, heightened inflammatory response, hyperkeratosis of the follicular epithelium, and colonization of sebaceous glands by the bacterium Cutibacterium acnes (formerly Propionibacterium acnes) (Moradi Tuchayi et al., 2015; Dreno et al., 2018; Luo et al., 2021). Sebaceous cells, the primary cells involved in acne formation, produce sebum lipids; high sebum levels foster an anaerobic, lipid-rich environment in the follicle. In this environment, C. acnes proliferates, exacerbating acne development (Clayton et al., 2019; Zouboulis et al., 2022). Additionally, the inflammatory microenvironment is modulated by the secretion of inflammatory cytokines (Farfán et al., 2022). As overactive sebaceous glands contribute to the pathological process of acne development, targeted destruction of sebaceous gland cells is an effective acne treatment.

Contemporary approaches to severe acne treatment include topical and oral medications, such as isotretinoin and oral antibiotics, respectively (Habeshian and Cohen, 2020). However, their prolonged use is constrained by systemic adverse effects (including skin, mucosal, and gastrointestinal inflammation). 5-aminolevulinic acid-photodynamic therapy (ALA-PDT) is a prevalent noninvasive treatment for severe acne vulgaris (Shi et al., 2022). However, ALA-PDT is associated with adverse effects, such as pain, erythema, burning of the skin, pruritus, and hyperpigmentation, limiting its clinical utility (Zhang et al., 2021). Furthermore, the hydrophilic nature of ALA limits its efficacy in PDT by restricting its tissue and cell membrane permeability.

Photothermal therapy (PTT) has been extensively employed in treating tumors and inflammatory diseases (Li et al., 2020). The superior photothermal conversion efficiency of nanoparticles in PTT, coupled with their targeted approach to specific lesions, minimizes damage to surrounding tissues. This, along with the advantage of shorter irradiation times, has garnered increasing research interest in nanoparticle-mediated PTT for skin diseases, particularly melanoma (Zeng et al., 2023). Presently, umbilical cord mesenchymal stem cell membranes (MSCM) are extensively investigated within the realm of drug delivery systems, attributed to their minimal immunogenic properties, targeted delivery capabilities, and readily accessible nature (Tanmoy et al., 2014). Recently, gold-NP-mediated PTT has demonstrated promise in preliminary human clinical trials for refractory acne (Suh et al., 2021; Seo et al., 2022). We previously demonstrated that coating Au-Ag-PDA NPs with umbilical cord mesenchymal stem cell membranes (MSCM) to form Au-Ag-PDA@MSCM NPs enhanced cellular uptake, improved NP dispersion and biocompatibility, and optimized PTT efficiency. Additionally, we observed that low-concentration Au-Ag-PDA@MSCM-mediated PTT can effectively reduce sebaceous gland tissues in golden hamsters and diminish sebum secretion without damaging the epidermis (Tianqi et al., 2020), However, the specific mechanisms by which nanoparticle-mediated PTT treats acne remain unexplored. Investigating these mechanisms is crucial for enhancing its clinical efficacy in acne management.

In this study, proteomic analysis revealed preliminary evidence of ferroptosis in the sebaceous gland tissues of golden hamsters treated with Au-Ag-PDA@MSCM-PTT. Furthermore, nanoparticles encapsulated with MSCM demonstrated robust efficacy both *in vitro* and *in vivo*. Ferroptosis represents a lipid peroxidation-mediated form of programmed cell death, where factors inducing cellular oxidative stress, such as heat, radiation, and redox dynamics disequilibrium, facilitate lipid oxidation modifications within the lipid bilayer. This process, with lipid peroxidation as a crucial regulator of cellular destiny, involves interconnected events such as the Fenton reaction post-iron ion accumulation, glutathione (GSH) depletion, dysregulation of the cystine/glutamate antiporter system xc-, peroxidation of polyunsaturated fatty acids (PUFAs) leading to compromised cellular membrane integrity, and reactive oxygen species (ROS) accumulation within mitochondria (Jiang et al., 2021). Consequently, in the course of cellular ferroptosis, a spectrum of biochemical changes ensues, characterized by significant glutathione (GSH) depletion, heightened concentration of sub-Fe ions, and a notable increase in malondialdehyde (MDA). Given that the accumulation of lipid reactive oxygen species (ROS) serves as a consequential outcome of lipid peroxidation and represents a hallmark of ferroptosis, such alterations are routinely observed. Furthermore, impairments in the mitochondrial respiratory chain, along with mitochondrial condensation and the disruption of mitochondrial cristae, serve as critical biomarkers for identifying ferroptosis in cellular structures.

In the present investigation, we employed a comprehensive approach incorporating proteomic analysis, alongside a suite of both *in vitro* and *in vivo* experimental techniques such as Western Blotting (WB), small interfering RNA (siRNA) interventions, and transmission electron microscopy, to preliminarily elucidate the mechanisms by which nanoparticle-mediated PTT induces ferroptosis to suppress lipid secretion in sebaceous glands. Initial findings underscore the pivotal role of Acyl-CoA synthetase long-chain family member 4 (Acsl4) in this bio-chemical pathway and demonstrate that encapsulation with MSCM markedly augments the effectiveness of PTT-induced ferroptosis in sebaceous gland cells. Given these insights, we contend that our research furnishes novel experimental evidence and a theoretical framework for employing PTT in the clinical treatment of acne.

## 2 Materials and methods

### 2.1 Preparation and characterization of mesenchymal stem cell membrane-coated Au-Ag-PDA NPs

The Au-Ag PDA NPs were synthesized following established procedures with minor modifications (Li et al., 2015). One hundred microliters (100 µL) of 100 mM HAuCl4 aqueous solution in 10 mL deionized water incubated with 1 mL of 1 mM Ag seeds for 2 min. Subsequently, 1 mL of 30 mM hydroquinone was added to the growth solution to obtain branched Au-Ag NPs. Further, 10 mL Tris-buffer solution and 400 µL of 30 mM dopamine solution were introduced into the branched Au-Ag NP solution. After 3 h of stirring at 27°C, the Au-Ag-PDA NPs were obtained. Human umbilical cord mesenchymal stem cell (MSC) lines, sourced from the China–Japan Union Hospital Scientific Research Centre, Jilin University (Changchun, China), provided MSC membrane (MSCM) vesicles as previously described (Gao et al., 2016). Briefly, we used a hypotonic solution to lyse the MSCs, followed by a freeze-thaw cycle at −80°C and subsequent differential centrifugation to obtain cell membranes. These MSC membranes were combined with Au-Ag-PDA NPs under sonication for 30 min to uniformly coat the Au-Ag-PDA NPs. Finally, the mixture was centrifuged to obtain Au-Ag-PDA@MSCM (Tianqi et al., 2020). TEM (JEM-1400; JEOL, Tokyo, Japan) was employed for observing NPs. Au-Ag-PDA NP solutions with concentrations of 20, 40, and 80 µg/mL were irradiated using an 808 nm near-infrared (NIR) LASER at a power of 1 W/cm^2^. Temperature changes in the solutions after 0, 2, 4, 6, 8, and 10 min of irradiation were recorded using an infrared imager (Supplementary Figure S1).

### 2.2 Cell culture and treatment

SZ95 human sebaceous gland cells (Zouboulis et al., 1999) were cultured in Dulbecco’s Modified Eagle’s Medium (DMEM) supplemented with 10% Fetal Bovine Serum (FBS) (Gibco, United States) and 100 U/mL each of penicillin and streptomycin (Gibco, United States). The culture was maintained in an incubator set at 37°C in an atmosphere of 95% air and 5% CO_2_. Six-well plates were seeded with 1 × 10^6^ cells per well, and 40 μg/mL of Au-Ag-PDA@MSCM NPs were prepared in a complete medium before being incubated with SZ95 cells at 37°C for 4 h. Following incubation, the six-well plates were removed from the incubator and exposed to 808 nm LASER irradiation (1 W/cm^2^, 8 min) once the medium reached room temperature (25°C). Subsequently, cells were harvested 8 h post-irradiation for WB; Fe^2+^ detection, GSH activity, MDA, lipid ROS assays; and Oil Red O staining. In the experimental setup involving the ferroptosis inhibitor, 15 nmol dosage of Ferrostatin-1 (S7243; Selleck Chemicals, Houston, TX, United States) was added to each well of the 6-well plate and incubated for 2 h prior to exposure to 808 nm wavelength laser irradiation.

Insulin-like growth factor-1 (IGF-1) enhances sebum secretion in SZ95 cells (Gu et al., 2022). Therefore, SZ95 cells were stimulated with 20 ng/mL recombinant human IGF-1 (HZ-1322, Proteintech, United States of America) for 24 h; the lipid accumulation was monitored using Oil Red O staining.

Acsl4 siRNA (50 nM) was transfected using the RFect siRNA transfection reagent (RFect: 11011; Shanghai Integrated Biotech Solutions Co., Ltd.), following the manufacturer’s instructions. The cells were then incubated for 48 h. Subsequently, SZ95 sebocytes were subjected to the aforementioned cellular treatments. The sequences of siRNA were as follows: Acsl4: Sense 5ʹ-GAG​GCU​UCC​UAU​CUG​AUU​ACC-3ʹ; Anti-sense 5ʹ-UAA​UCA​GAU​AGG​AAG​CCU​CAG-3ʹ. Negative control: Sense 5ʹ-UUC​UCC​GAA​CGU​GUC​ACG​UTT-3ʹ; Anti-sense 5ʹ-ACG​UGA​CAC​GUU​CGG​AGA​ATT-3ʹ.

### 2.3 *In vivo* PTT

All animal studies were conducted according to the UK Animal Welfare Act 1986 and associated guidelines, the EU Animal Welfare Directive 2010/63/EC, or the National Institutes of Health (NIH) Guide for the Care and Use of Laboratory Animals. The study protocol was reviewed and approved by the Ethics Review Committee for Animal Experiments, School of Basic Medical Sciences, Jilin University (No. 2023–229). Forty male golden hamsters (Liaoning Changsheng Biotechnology Co., Ltd., Shenyang, China), weighing 80–120 g, were used in this study.

Liquid chromatography-mass spectrometry (LC-MS) was utilized for proteomics analysis. DEPs were identified as those meeting the criteria of a fold change (FC) > 1.2, with *p* < 0.05 indicating statistical significance. Based on previous *in vivo* experiments and grouping methods, optimal parameters for *in vivo* studies were determined: NP concentration of 20 μg/mL and 808 nm LASER irradiation at 0.5 W/cm^2^ for 3 min (Tianqi et al., 2020). The experimental procedure is presented in Supplementary Figure S3. Sebaceous gland tissue samples were collected from golden hamster flank organs and divided into four groups: A, blank sebaceous gland tissue group (Control, *n* = 5); B, sebaceous gland tissue group post-C. acnes inoculation (C. acnes, *n* = 5); C, sebaceous gland inflammatory acne model group post-application of Au-Ag-PDA@MSCM and 808 nm LASER irradiation (C. acnes + Au-Ag-PDA@MSCM, *n* = 5); and D, sebaceous gland acne model group post-application of Au-Ag-PDA NPs and 808 nm LASER irradiation (C. acnes + Au-Ag-PDA, *n* = 5). Our prior findings revealed a substantial decrease in the size of sebaceous gland tissue and a reduction in sebum secretion on day 20 post-PTT (Tianqi et al., 2020). Consequently, this time point was selected for the LC-MS analysis of the golden hamster sebaceous gland tissue. The analysis was replicated thrice across each of the four designated groups, amassing a total of 12 samples per iteration.

To elucidate the direct suppression of sebum production in sebaceous glands mediated by Au-Ag-PDA@MSCM through PTT, C. acnes was omitted from established experimental protocols. Subsequently, 30 μL of a 20 μg/mL Au-Ag-PDA@MSCM NP solution was topically applied to the sebaceous gland tissue of the golden hamster flank organs to discern the specific effects of PTT on sebaceous gland functionality. Subsequently, the area was irradiated with an 808 nm LASER (0.5 W/cm^2^, 3 min). Following the therapeutic intervention, sebaceous gland tissues were meticulously procured for comprehensive histological evaluation using HE and Oil Red O staining, as well as ultrastructural examination using TEM. These assessments were conducted at the following pivotal junctures: blank control group (*n* = 5), immediately post-treatment (*n* = 5), and followed by evaluations on days 3 (*n* = 5) and 5 (*n* = 5).

### 2.4 Protein extraction and enzymatic hydrolysis using trypsin

Tissue samples were scraped from glass slides, transferred to 1.5 mL centrifuge tubes, and disrupted using sonication with four volumes of lysis buffer (1% sodium dodecyl sulfate [SDS], 1% protease inhibitor). The samples were centrifuged at 12,000 × g for 10 min at 4°C to remove debris, and the resulting supernatants were transferred to new centrifuge tubes. A bicinchoninic acid (BCA) protein assay kit was used to determine the protein concentrations. After adding 200 mM triethylammonium bicarbonate at a ratio of 1:50 (w/w) and sonicating the protein mixture, the proteins were digested overnight using trypsin. After a 30-min reduction at 56°C, 5 mM dithiothreitol was added to the mixture. Iodoacetamide was then added to a final concentration of 11 mM, and the mixture was incubated in the dark for 15 min. A C18 solid-phase extraction column was used to desalt the peptides.

### 2.5 Liquid chromatography-mass spectrometry (LC-MS) analysis

The peptides were resolved using a NanoElute ultra-performance liquid chromatography system after solubilization in LC mobile phase A (0.1% formic acid and 2% acetonitrile in water). Mobile phase B consisted of 0.1% formic acid and 100% acetonitrile. The gradient used was: 0–70 min, 6%–24% B; 70–84 min, 24%–35% B; 84–87 min, 35%–80% B; 87–90 min, 80% B. The flow rate was maintained at 450 nL/min. A capillary ion source was used to ionize the peptides, and timsTOF Pro (Bruker, Billerica, MA, United States) was used for MS analysis. The peptide parent ion and its secondary fragments were detected and analyzed using high-resolution time-of-flight MS, with the ion source voltage set to 2.0 kV. Secondary mass spectra were obtained between 100 and 1,700. The data were acquired using parallel cumulative serial fragmentation (PASEF). A primary mass spectrum was acquired, and 10 acquisitions were obtained in the PASEF mode to obtain a secondary spectrum, with parent ion charges in the range of 0–5. The dynamic exclusion time for the tandem MS scan was set to 30 s to avoid repetitively scanning the parent ion. The results were considered significant when the relative protein expression ratio threshold increased by ≥ 1.2-fold or decreased by ≥ 0.6-fold. The mass spectrometry proteomics data have been deposited into the ProteomeXchange Consortium (http://proteomecentral.proteomexchange.org) via the iProX partner repository with the dataset identifier PXD046301.

### 2.6 Functional and pathway enrichment analysis of proteins

Bioinformatics analyses were performed on each of the four groups outlined in Section 4.2 (Control, C. acnes, C. acnes + Au-Ag-PDA@MSCM, and C. acnes + Au-Ag-PDA), and the results were compared. The DEPs in each group were functionally annotated (BPs, cellular components, and MFs) using the GO annotation database (EMBL-EBI, Hinxton, Cambridgeshire, UK). The KEGG pathway database was employed to identify the signaling pathways involved. Fisher’s exact test was used to calculate the *p* values (*p* < 0.05 was considered significant). Data from all categories obtained after enrichment were collected, and categories with a *p*-value of at least 0.05 in at least one cluster were retained. This filtered *p*-value matrix was transformed using the function x = −log10 (*p*-value). Subsequently, the x values were z-transformed for each functional category, and the z-scores were clustered by gene using one-way hierarchical clustering (Euclidean distance and average linkage clustering). Heatmaps were generated using the R package “heatmap” to visualize the cluster membership.

### 2.7 HE and ORO staining

Sebaceous gland tissues treated with PTT were collected at different time points (days 0, 3, and 5). These tissues were postfixed in formalin and embedded in paraffin for tissue blocks. Subsequently, tissue blocks were sectioned into thin slices.First, the sections were immersed in a staining agent, such as an oxidizing agent for hematoxylin dye (hematoxylin), to stain the nuclei. The sections were then stained with an acidic dye, such as an acidic eluent (eosin), to stain the cytoplasmic structures. Alternatively, the sections were immersed in ORO stain, a lipophilic dye that selectively stains lipids red. Stained tissue sections were dehydrated in different alcohol concentrations and cleaned with the appropriate solvents. Finally, the sections were covered with a clearing agent (benzene or xylene) and placed on a slide.

For ORO staining, the experimental protocol was performed using the Oil Red O Stain Kit for Cultured Cells (G1262; Solarbio, Beijing, China). In accordance with the provided guidelines, cells were fixed in ORO fixative solution for 20–30 min. After fixation, the sections were thoroughly rinsed with physiological saline and immersed in 60% isopropanol for 5 min. After isopropanol removal, ORO stain was applied for an optimal staining period of 10–20 min. Following a double wash with physiological saline, Mayer’s hematoxylin staining solution was added to each well for 1–2 min to counterstain the nuclei. Finally, ORO buffer was systematically applied to each well and incubated for 1 min. Observation and image capture were performed using a microscope.

### 2.8 Live and dead cell staining

The Viability/Cytotoxicity Assay Kit for Animal Live and Dead Cells (Calcein AM, EthD-I) (PF00008; Proteintech, Rosemont, IL, United States) was used to prepare a staining working solution containing 2 µM Calcein AM and 4 µM EthD-I in accordance with the manufacturer’s instructions. For adherent cells, an ample volume of Calcein AM/EthD-I staining working solution was added, followed by incubation at room temperature in the dark for 15–20 min. The labeled cells were observed under a fluorescence microscope.

### 2.9 Fe^2+^ measurements

Fe^2+^ levels in sebaceous gland tissues were measured using an Iron Assay Kit (K390-100; BioVision, Milpitas, CA, United States). Adherent cells (SZ95 sebocytes) were immediately homogenized in Fe^2+^ assay buffer and centrifuged. The supernatant was collected, and the assay was performed according to the manufacturer’s instructions.

### 2.10 Measurement of MDA levels

The MDA levels in SZ95 sebocytes were measured using an MDA assay kit (A003-1-1; Nanjing, China). Fresh sebaceous gland tissue or adherent cells (SZ95 sebocytes) were immediately homogenized in phosphate-buffered saline (PBS). After centrifugation, the supernatants were collected. Various reagents were added depending on the procedure used. The optical density of each well was measured at 532 nm using a microplate reader.

### 2.11 GSH activity assay

Ferroptosis is triggered by disruption of the cellular redox balance, and GSH plays a significant role in mitigating the accumulation of lipid ROS. Ferroptosis occurs when GSH is depleted in cells (Ursini and Maiorino, 2020). GSH assay kits from the Nanjing Jiancheng Bioengineering Institute (A006-2-1; Nanjing, China) were used to detect the reduced GSH levels. Fresh sebaceous gland tissue or adherent cells (SZ95 sebocytes) were immediately homogenized in PBS. The supernatant was subjected to a colorimetric assay involving a 5-min color development at an optical wavelength of 420 nm, followed by quantification of the absorbance values for each sample.

### 2.12 Investigation of sebaceous gland tissue using Bio-TEM

Following treatment with Au-Ag-PDA@MSCM NPs (20 µg/mL) and irradiation with an 808 nm LASER (0.5 W/cm^2^, 3 min), sebaceous gland tissue samples were promptly collected, both immediately and on day 5 post-irradiation. The tissue samples were fixed in 2.5% electron-microscopy-grade glutaraldehyde at 4°C overnight. Subsequently, the sections were stained with 4% osmium tetroxide for 30 min at room temperature. Dehydration was performed at room temperature in an ethanol gradient (30%–100%); the samples were then embedded in epoxy and cured at 60°C for 48 h. Ultrathin tissue sections (50–70 nm) were stained with 5% uranyl acetate and 2% lead citrate before TEM analysis (JEM 2100; JEOL).

### 2.13 Lipid peroxidation and analysis

Cells were cultured in 15-mm confocal dishes at a density of 1 × 10^5^ cells/well. They were subsequently subjected to Au-Ag-PDA@MSCM-mediated PTT. The dishes were then rinsed with PBS and incubated with 2 mM BODIPY 581/591 C11 (D3861; Invitrogen, Waltham, MA, United States) in PBS for 20 min to assess lipid peroxidation levels. Following this, the samples were visualized using a confocal microscope (Carl Zeiss, Jena, Germany), and fluorescence was quantitatively analyzed using ImageJ software.

### 2.14 Western blotting analysis

Sebaceous gland tissues and adherent SZ95 sebocytes were homogenized and lysed in radioimmunoprecipitation assay buffer. The concentration of the collected lysate was determined using BCA assay. The expression levels of Acsl4, large tumor suppressor 1 and 2 (p-LATS1/2), yes-associated protein (p-YAP), glutathione peroxidase 4 (GPX4), and solute carrier family 7 member 11 (SLC7A11) in cell lysates (30 µg) were determined using WB. The samples underwent thermal denaturation in Laemmli buffer containing 4% SDS at 100°C for 5 min. Proteins were resolved on a 12% sodium dodecyl sulfate–polyacrylamide electrophoresis (SDS–PAGE) gel (P0012A; Beyotime, Nantong, China) for 60 min at 110 V. Subsequently, proteins were transferred onto polyvinylidene fluoride membranes using the semi-wet transfer method at 220 mA for 120 min. The membranes were blocked with 5% (w/v) bovine serum albumin and 0.1% (v/v) Tween-20 for 1 h at room temperature. Antibodies against Gpx4 (67763-1-Ig; Proteintech, San Diego, CA, United States), SLC7A11 (26864-1-AP, Proteintech), Acsl4 (66617-1-Ig, Proteintech), LATS1/2 (DF7517; Affinity Biosciences, Cincinnati, OH, United States), phospho-LATS1 + LATS2 (Thr1079 + Thr1041; Bioss, Woburn, MA, United States), YAP (Ser127) (AF3328, Affinity Biosciences) were added to the blocking buffer, and the membranes were incubated overnight at 4°C. This was followed by incubation with a secondary antibody (HAF008; 1:2,000 dilution; R&D Systems, Minneapolis, MN, United States) for 1 h at room temperature. Blotted proteins were visualized using an Odyssey infrared imaging system (LI-COR Biosciences, Lincoln, NE, United States).

### 2.15 Quantitative reverse transcription PCR (qRT-PCR)

RNA was isolated from SZ95 sebocytes using the EZ-press RNA Purification Kit (B0004DP, EZBioscience, Roseville, MN, United States). Gene expression was analyzed using ToloBio kits (Universal, #22204; #22107; Shanghai, China). The primer sequences used were as follows: human Acsl4 F: 5ʹ-CAT​CCC​TGG​AGC​AGA​TAC​TCT-3ʹ; Homo Acsl4 R: 5ʹ-TCA​CTT​AGG​ATT​TCC​CTG​GTC​C-3ʹ. Homo GAPDH F: 5ʹ-CAT​GAG​AAG​TAT​GAC​AAC​AGC​CT-3ʹ; Homo GAPDH R: 5ʹ-AGT​CCT​TCC​ACG​ATA​CCA​AAG​T-3ʹ. After reverse transcription, the q-PCR reaction involves three stages: Stage 1, pre-denaturation at 95°C for 30 s; Stage 2, amplification consisting of 40 cycles of 95°C for 10 s and 60°C for 30 s; and Stage 3, the melt curve analysis, performed according to the instrument’s default program.

## 3 Results

### 3.1 Characterization of Au-Ag-PDA@MSCM NPs

**FIGURE 1:**
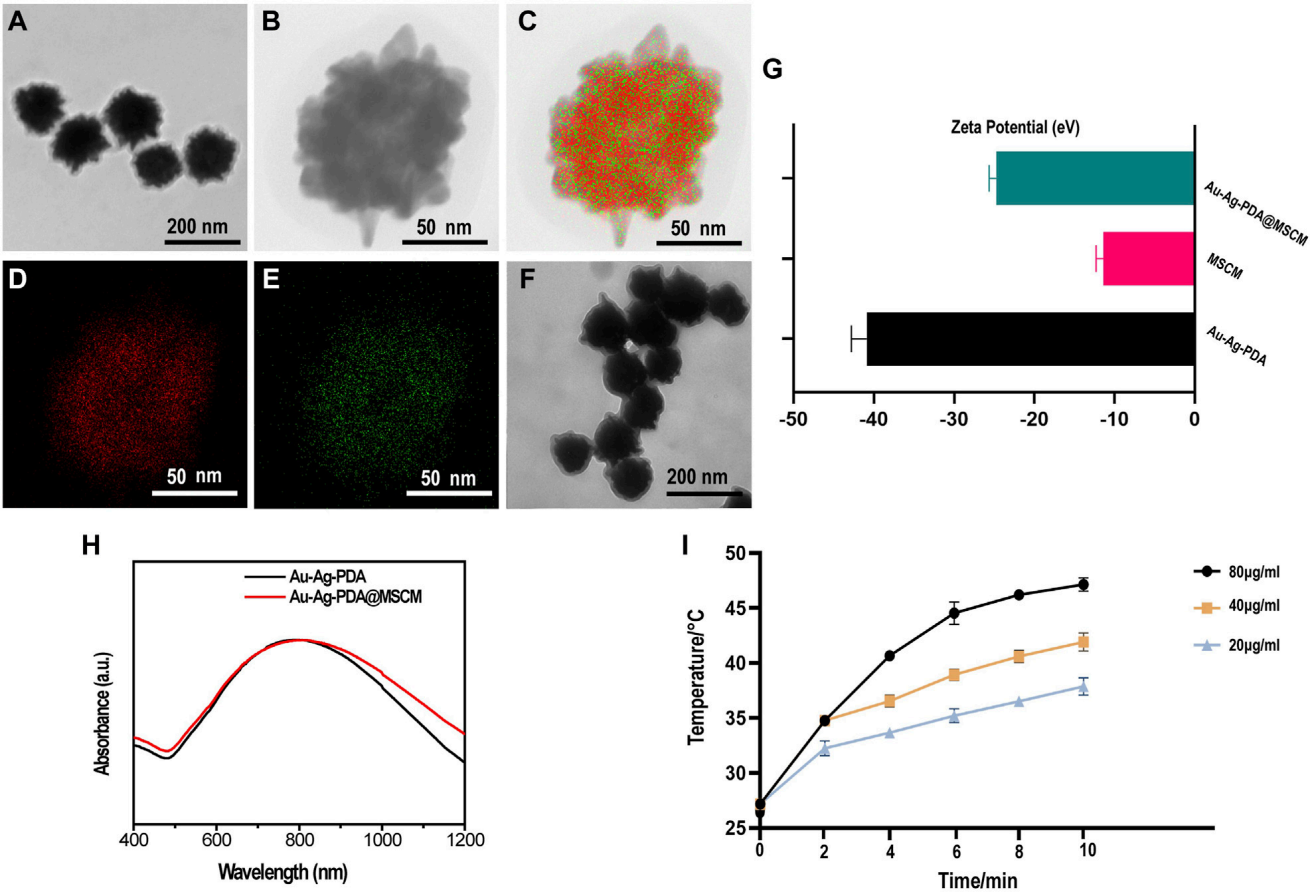
Characterization of Au-Ag-PDA@MSCM NPs. Transmission electron microscope images of Au-Ag-PDA NPs **(A)** and Au-Ag-PDA@MSCM **(F**). HRTEM image **(B)** and HAADF-STEM-EDS mapping images of Au element **(D)**, Ag element **(E)** and overlapping images **(C)** of Au-Ag-PDA NPs. **(G)** Zeta potential of Au-Ag-PDA, MSCM and Au-Ag-PDA@MSCM. **(H)** UV-vis spectra of the Au-Ag-PDA and Au-Ag-PDA@MSCM. **(I)** Temperature record of the Au-Ag-PDA@MSCM with different concentrations under 808 nm LASER irradiation at 1 W/cm^2^.

The synthesized Au-Ag-PDA NPs had a diameter of 140–160 nm (Figure 1A). High-angle annular dark-field imaging (HAADF)-scanning transmission electron microscopy (STEM) and energy-dispersive X-ray spectroscopy (EDS) elemental mapping images revealed the presence of Au and Ag in the Au-Ag-PDA NPs (Figures 1B–E). Subsequently, the Au-Ag-PDA NPs were coated with MSCMs (Figure 1F). The zeta potential of Au-Ag-PDA@MSCM was determined to validate the coating, and the results showed zeta potentials of −40.86 eV for Au-Ag-PDA NPs, −11.4 eV for MSCM, and −24.73 eV for Au-Ag-PDA@MSCM (Figure 1G). This suggests successful binding of MSCMs to the surface of Au-Ag-PDA NPs. UV-vis absorbance demonstrated that the MSCM coating maintained a high absorption peak at approximately 808 nm, indicating that Au-Ag-PDA@MCSM retained excellent photothermal conversion properties (Figure 1H). The photothermal properties of Au-Ag-PDA@MSCM at varying concentrations (20, 40, and 80 µg/mL) were evaluated under 808 nm NIR laser irradiation. (Supplementary Figure S1) presents the temperature profiles of the nanoparticle solutions captured by an infrared imager. Figure 1I illustrates the temperature variation curves, indicating that the temperature of the nanoparticle solutions increases with both concentration and irradiation time. This confirms their sustained excellent photothermal conversion ability.

### 3.2 Protein identification and quantification

**FIGURE 2:**
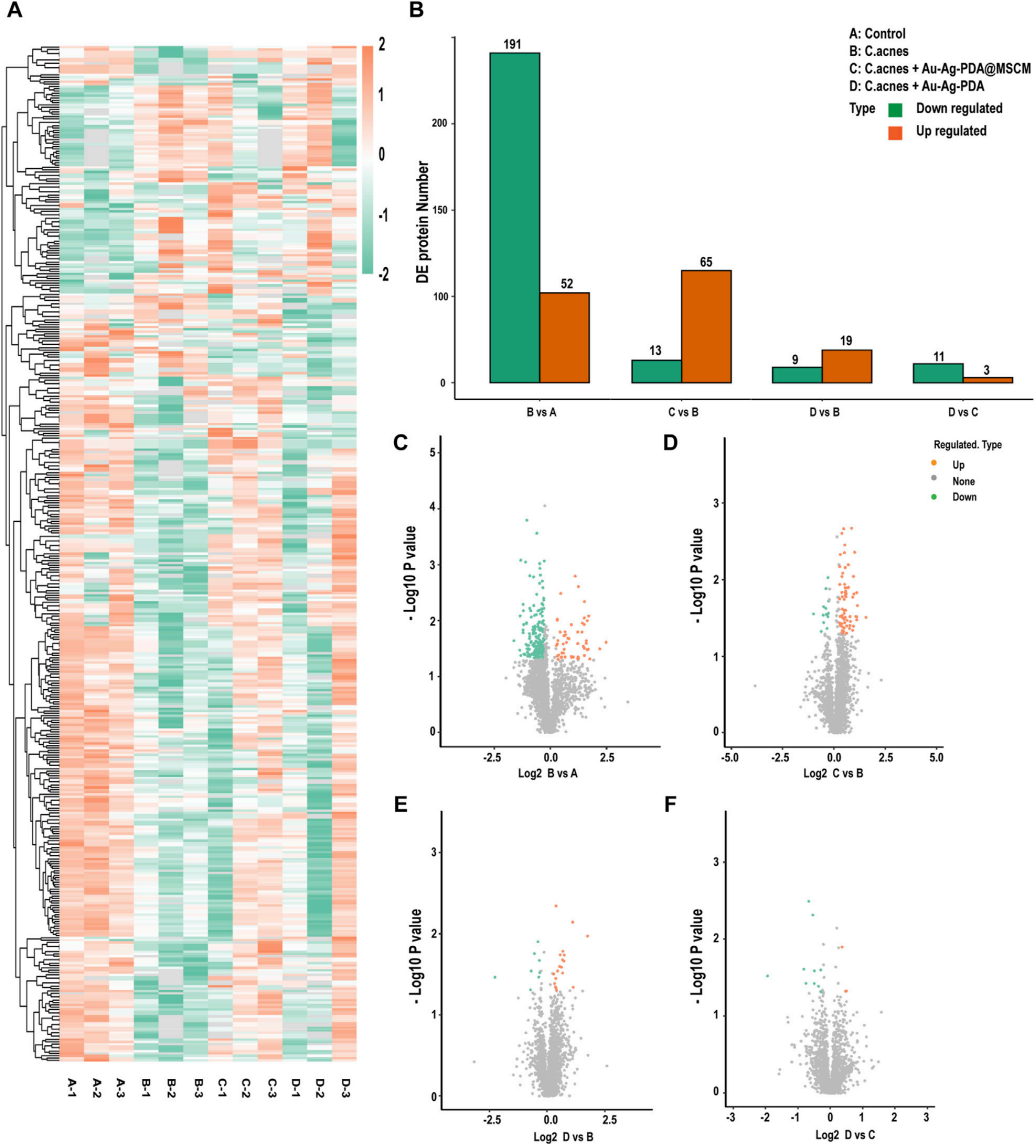
Protein identification and quantification. Distribution of sebaceous gland protein data for the four groups. **(A)** Differentially expressed (DE) protein heatmap. **(B)** Histogram of the number of differentially expressed proteins per group. **(C–F)** Volcano plots of DE proteins. The horizontal axis shows the fold change (log2 transformed) and the vertical axis shows the *p*-value (log10 transformed). The red dots indicate upregulated proteins and the green dots indicate downregulated proteins.

Sebaceous gland tissue samples from the four groups (A: Control; B: C. acnes; C: C. acnes + Au-Ag-PDA@MSCM; D: C. acnes + Au-Ag-PDA) were trypsinized and analyzed using LC-MS (Supplementary Figure S2A). The course of the *in vivo* experiments is presented in Supplementary Figure S3. A total of 3,211 proteins (Figure 2A), meeting the threshold criteria of fold change (FC) > 1.2 and a *p*-value <0.05, were identified). In Group B, 243 proteins differentially expressed compared with those in Group A (Figures 2B,C). Most of the upregulated proteins, including Atp1b1 (A0A1U7QRD1), LOC110341478 (A0A3Q0CVQ9), Myl2 (A0A1U7QTQ7), Casq2 (A0A1U7QD56), and Tnnc1 (A0A1U7RBF6), were primarily involved in the regulation of endoplasmic reticulum calcium ion homeostasis. C compared with Group B, Group C revealed enrichment of 78 DEPs, with 65 upregulated and 13 downregulated proteins (Figures 2B,D). Notably, Acsl4 (A0A1U8CEC2), a protein involved in mediating cellular ferroptosis, was upregulated, though not statistically significant (Table 1). Downregulated proteins were associated with oxidative phosphorylation and mitochondrial respiration. For example, Ndufv2 (A0A3Q0CXF4) and LOC101843749 (A0A1U7QK94) were downregulated by 0.76-fold and 0.69-fold, respectively (Table 1). In the comparison between Groups D and B, 28 DEPs were identified; among these, 19 DEPs were upregulated, and 9 DEPs were downregulated proteins (Figures 2B,E). Similar to the DEPs between Groups C and B, Acsl4 (A0A1U8CEC2), a protein involved in ferroptosis, was upregulated in Group D. As Groups D and C underwent similar treatments, a smaller number of DEPs were observed between the two groups (Figures 2B,F).

**TABLE 1:** Differentially expressed proteins in the ferroptosis, Hippo, oxidative phosphorylation, and TCA cycle pathways before and after Au-Ag-PDA@MSCM photothermal therapy intervention.

Pathways	Protein accession number	Protein description	Gene name	C/B ratio	*p*-Value
ferroptosis	A0A1U8CEC2	acyl-CoA synthetase long-chain family member 4	Acsl4	1.67	0.99
	A0A3Q0CM78	heat-shock protein 90-alpha A1	Hsp90aa1	1.46	0.031
TCA cycle (map00020)	A0A1U7R004	fumarate-hydratase, mitochondrial	Fh	0.83	0.018
	P86225	isocitrate dehydrogenase (NAD) subunit alpha, mitochondrial	Idh3A	0.70	0.036
Oxidative phosphorylation (map00190)	A0A1U7Q3R5	cytochrome b-c1 complex subunit Rieske, mitochondrial	LOC101839974	0.82	0.027
	A0A1U7QK94	cytochrome c oxidase subunit 5A, mitochondrial	LOC101843749	0.69	0.029
	A0A3Q0CXF4	NADH dehydrogenase (ubiquinone) flavoprotein 2, mitochondrial	Ndufv2	0.76	0.013
Hippo signaling pathway (map04390)	A0A1U7R6Z8	14-3-3 protein eta	Ywhah	0.77	0.028
	A0A1U8BRN3	glycogen synthase kinase-3 beta	Gsk3b	1.21	0.012
	A0A1U8CE17	catenin beta-1	Ctnnb1	1.44	0.015

Notes: C/B ratio: DE, protein ratio between groups C (C. acnes + Au-Ag-PDA@MSCM) and B (C. acnes).

### 3.3 Gene ontology (GO) enrichment analysis of DEPs and kyoto encyclopedia of genes and genomes (KEGG) signaling pathways

**FIGURE 3:**
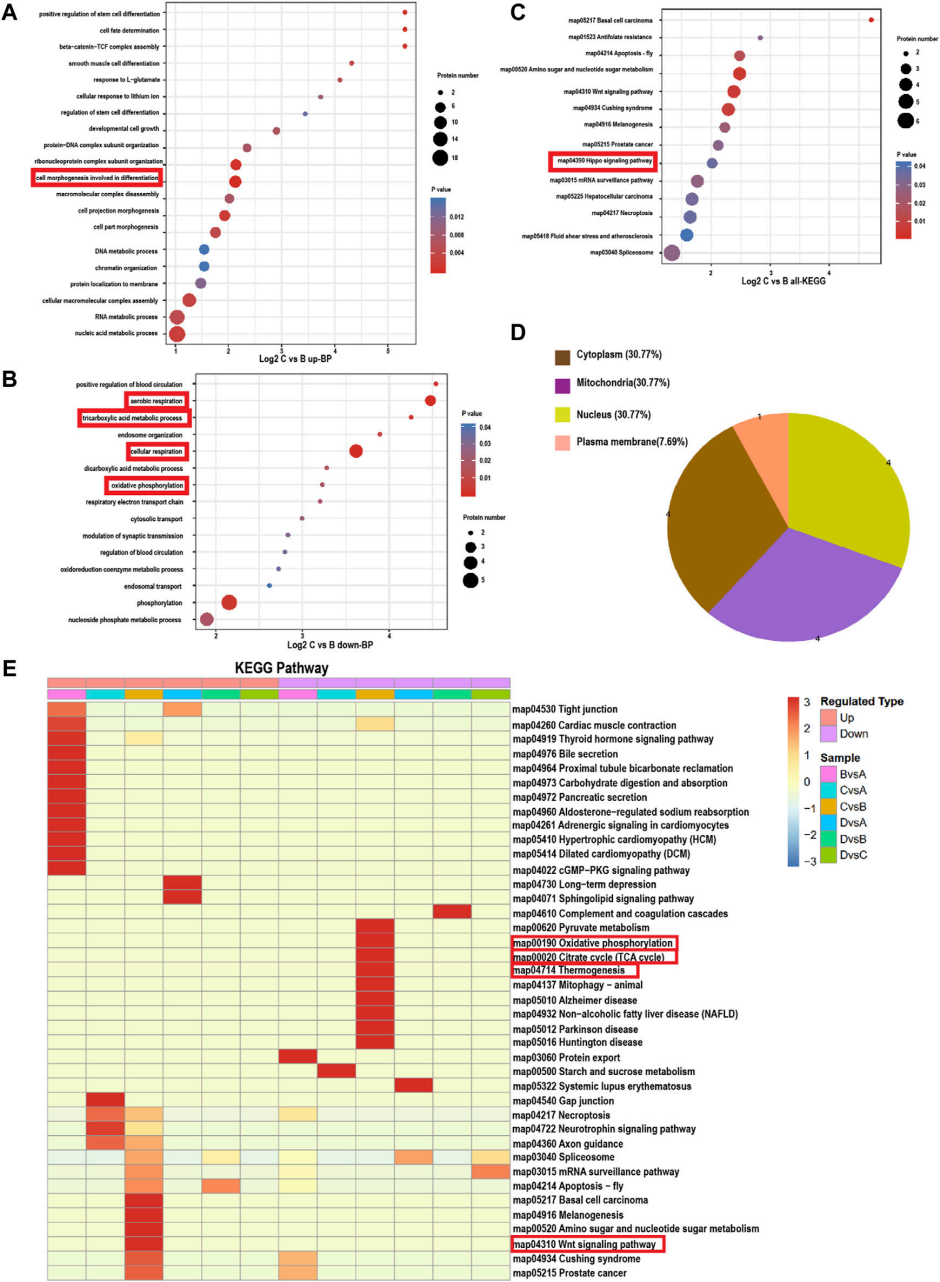
Bubble map based on bioprocess gene ontology enrichment classification and KEGG signaling pathway (Panel C: C. acnes + Au-Ag-PDA@MSCM; Panel B: C. acnes). **(A)** Biological processes involved in the upregulated proteins in group C compared to group **(B)**. **(B)** Biological processes involved in the downregulated proteins in group C compared to group **(B)**. Bubble chart of DE proteins enriched in Kyoto Encyclopedia of Genes and Genomes (KEGG) pathways. **(C)** KEGG pathways involved in all DE proteins in group C compared to group **(B)**. The bubble size indicates the number of DE proteins in the enriched pathway terms, and the bubble color indicates the *p*-value. **(D)** Subcellular locations of the proteins downregulated in group C compared to group **(B)**. **(E)** Heat map of the cluster analysis based on the Gene Ontology enrichment classifications of KEGG signalling pathway. The horizontal direction indicates the grouping and the vertical direction indicates the described functions. Red indicates a high degree of enrichment.

To further elucidate the molecular mechanisms underlying the ability of Au-Ag-PDA@MSCM-mediated PTT to inhibit sebum secretion, we utilized the GO database to analyze the DEPs in each group. The biological processes (BPs), cellular components (CCs), and molecular functions (MFs) associated with the DEPs were investigated (Figure 3; Supplementary Figures S2C, D). In the comparison between Groups A and B, upregulated proteins were implicated in inflammation-related BPs, including cation homeostasis (GO:0055080), calcium ion homeostasis (GO:0055074), and regulation of blood circulation (GO:1903522). One downregulated protein was associated with protein localization to the endoplasmic reticulum (GO:0070972) BP (Supplementary Figure S2B). In the comparison between Groups C and B, we observed that the primary BP associated with upregulated proteins was cell morphogenesis, linked to cell differentiation (GO:0000904; Figure 3A). Downregulated proteins were mainly associated with cellular respiration (GO:0045333), aerobic respiration (GO:0009060), oxidative phosphorylation (GO:0006119), and tricarboxylic acid metabolic process (GO:0072350; Figure 3B). Subcellular localization analysis revealed that 30.77% of these downregulated proteins were concentrated in the mitochondria (Figure 3D). Au-Ag-PDA@MSCM-mediated PTT diminished the energy production of sebaceous gland cells, leading to cell death. In the comparison between Groups D and B, the downregulated proteins were primarily enriched in the regulation of complement activation (GO:0030449) and regulating inflammatory response (GO:0050727) BPs (Supplementary Figure S2B), but not in BPs associated with the disruption of cellular function.

Next, we analyzed the DEPs in the sebaceous gland tissues of the four groups using the KEGG pathway database (Figure 3E). In the comparison between Groups B and A, we observed that the upregulated proteins were enriched in the calcium (map04020) and cAMP (map04024) signaling pathways, whereas downregulated proteins were mainly involved in the protein export (map03060) and protein processing in the endoplasmic reticulum (map04141) pathways (Figure 3E). When comparing Groups C and B, the DEPs were enriched in the Wnt (map04310) and Hippo (map04390) signaling pathways (Figure 3C). In contrast, downregulated proteins were enriched in the oxidative phosphorylation (map00190), thermogenesis (map04714), and citrate cycle (map00020) pathways (Figure 3E). Finally, the comparison between Groups D and B revealed that the downregulated proteins were enriched in the complement and coagulation cascades (map0461; Figure 3E).

### 3.4 PTT induces ferroptosis in SZ95 cells and inhibits IGF-1-stimulated sebum secretion

PTT has been investigated for its capacity to induce various forms of cell death, such as apoptosis and necroptosis, in tumor cells (Moros et al., 2020). Our proteomic analysis indicated that Ag-PDA@MSCM-mediated PTT compromised the integrity of the mitochondrial respiratory chain, culminating in the suppression of aerobic respiration (Table 1; Figure 3). Cells undergoing ferroptosis typically exhibit mitochondrial constriction, characterized by reduced cristae, dissipation of mitochondrial membrane potential, and increased mitochondrial membrane permeability, indicative of mitochondrial dysfunction (Dixon Scott et al., 2012). Consequently, we hypothesized that Ag-PDA@MSCM-mediated PTT could directly induce ferroptosis in the sebaceous gland tissues, reducing gland size and suppressing sebum secretion. Cellular heat stress responses commence at proximal temperatures of 41°C during PTT (The heat shock response, 2022).

**FIGURE 4:**
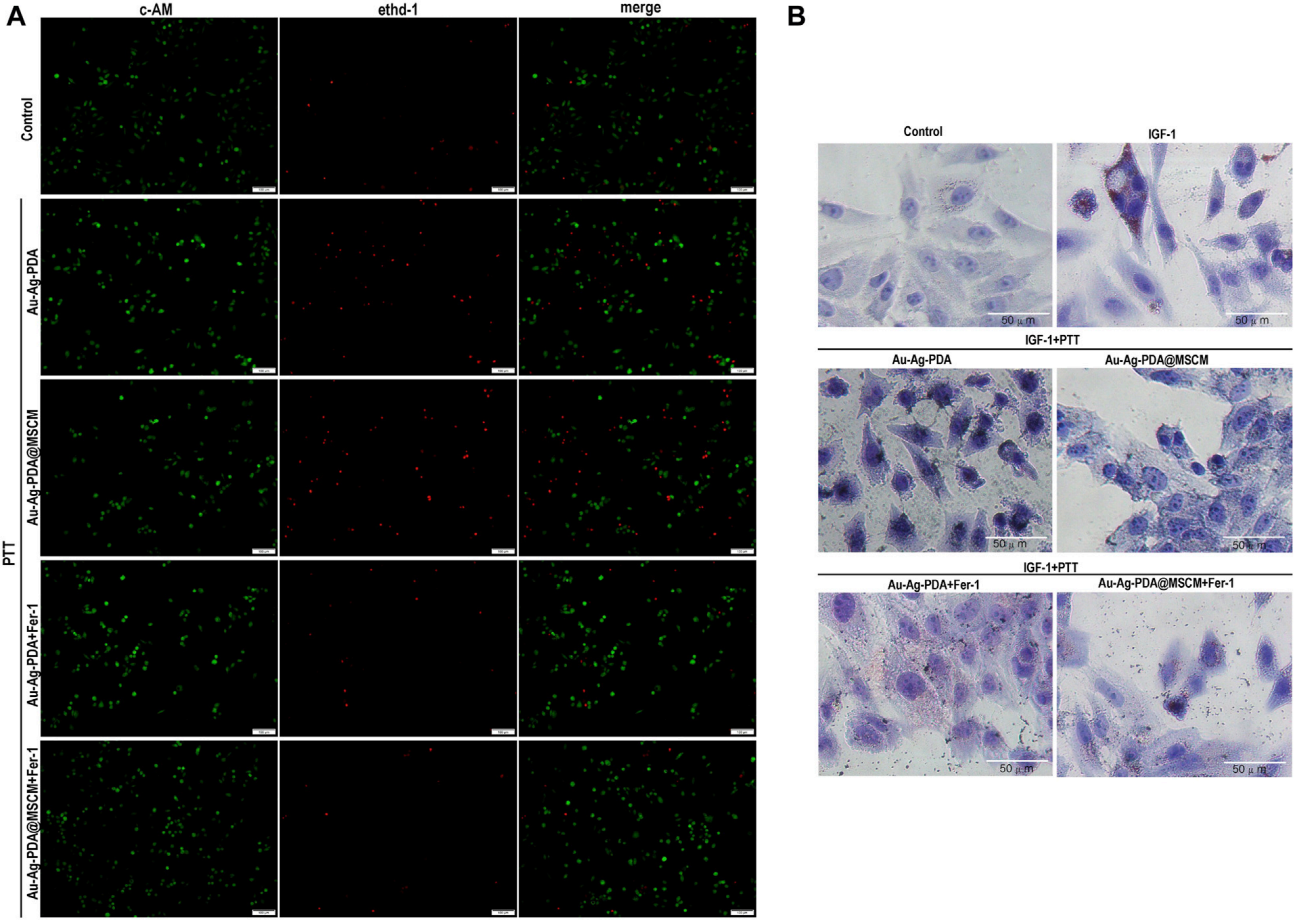
PTT induces ferroptosis in SZ95 cells and inhibits IGF-1-stimulated sebum secretion. In the presence of the ferroptosis inhibitor Fer-1, we evaluated the impact of PTT on the viability and sebum secretion of SZ95 cells. **(A)** Using fluorescence microscopy, we conducted live-dead cell staining; live cells were identified by green fluorescence, while dead cells were indicated by red fluorescence. **(B)** Following 24 h stimulation with IGF, Oil Red O staining was performed to visualize sebum secretion in SZ95 cells, denoted by red particles. Additionally, in the Au-Ag PDA and Au-Ag PDA@MSCM-mediated PTT treated groups, black particles signified the clustered nanoparticles. Microscope magnification is × 20, Scale bar: 50 μm.

To substantiate PTT-induced ferroptosis in SZ95 sebaceous gland cells, we employed 40 µg/mL nanoparticle concentrations and 1 W/cm^2^ laser irradiation over 8 min, thereby elevating the solution’s temperature to 40°C–41°C for PTT. We used Ferrostatin-1 (Fer-1), a ferroptosis inhibitor. Viability assays utilizing calcein acetoxymethyl ester (Calcein AM) and ethidium homodimer-1 (EthD-I) for live/dead cellular staining indicated living cells in green and dead cells in red (Figure 4A). Supplementary Figure S4 presents the statistical analysis of the percentage of live and dead cells in each group using ImageJ. Our findings revealed that both Au-Ag-PDA and Au-Ag-PDA@MSCM-mediated photothermal activity augmented the prevalence of red-stained cells, with a notably higher cell death rate in the Au-Ag-PDA@MSCM group. Notably, Fer-1 significantly mitigated post-PTT cell death.

Our analysis using Oil Red O (ORO) staining indicated a marked increase in lipid accumulated in SZ95 cells 24 h post-IGF-1 stimulation (Figure 4B). After Au-Ag-PDA- and Au-Ag-PDA@MSCM-mediated PTT, reductions in red lipid droplets and aberrations in cell morphology were observed in sebaceous gland cells. Furthermore, introducing the ferroptosis inhibitor Fer-1 substantially ameliorated these changes, indicating improved lipid droplet accumulation and cellular morphology.

### 3.5 PTT triggers ferroptosis in sebaceous gland cells by modulating Acsl4

Acsl4, a member of the long-chain acyl-CoA synthase (ACSL) family, plays a vital role in fatty acid metabolism and functions as an enzyme in lipid peroxidation, a fundamental reaction in cellular metabolism (Dang et al., 2022). Elevated temperatures stabilize Acsl4 expression, inducing ferroptosis in glioma cells, as well as causing alterations in mitochondrial morphology and membrane potential (Miao et al., 2022).

**FIGURE 5:**
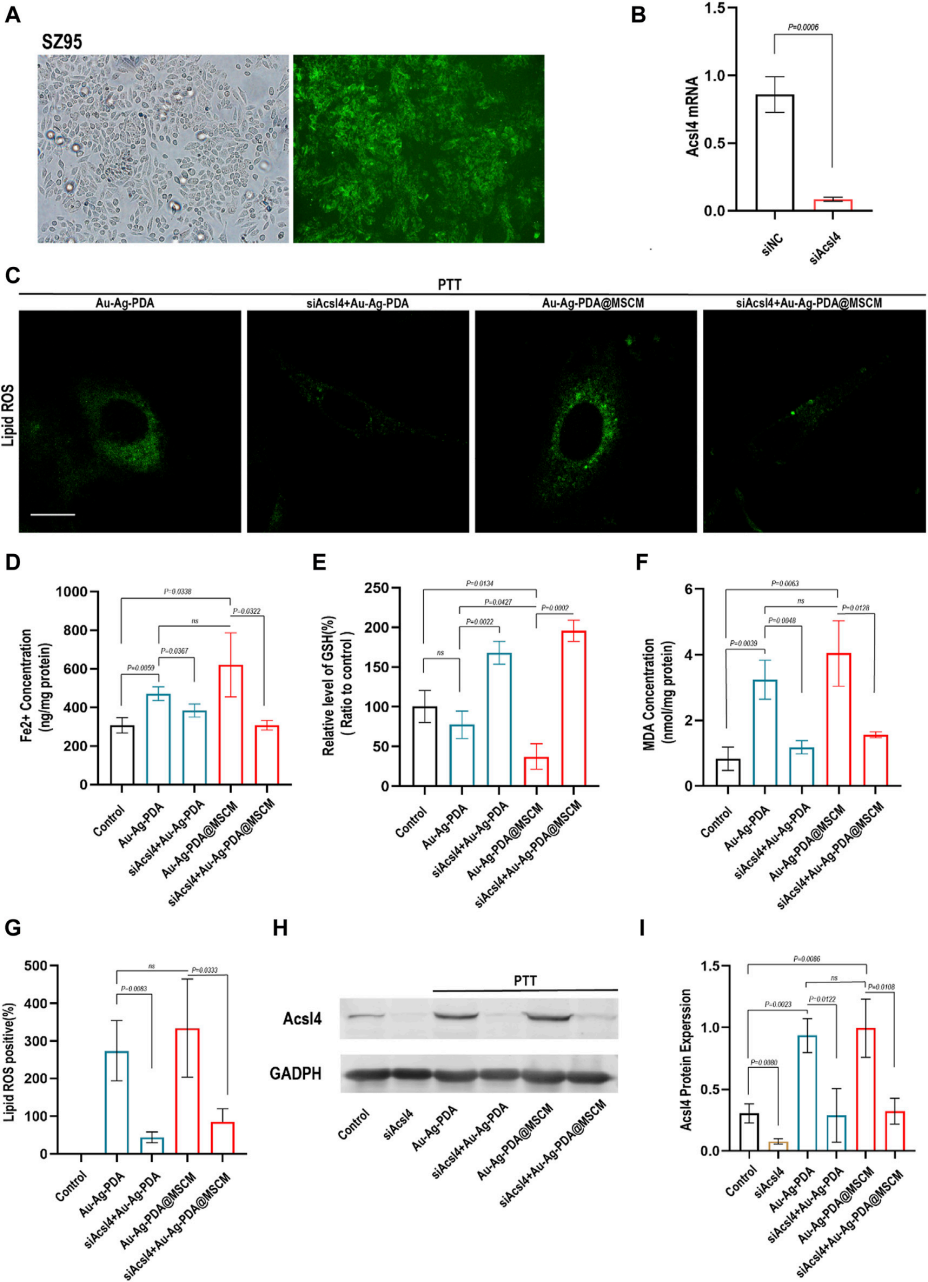
PTT triggers ferroptosis in SZ95 by modulating Acsl4. **(A)** Fluorescence microscopy images demonstrate the successful transfection of SZ95 sebocytes with green fluorescent-labeled siRNA (si-fam) at a concentration of 50 nm. The fluorescence microscopy images would show the presence of green fluorescence signal within the cells, indicating the uptake and intracellular localization of the siRNA. **(B)** Quantitative polymerase chain reaction (qPCR) was employed to detect the expression levels of the Acsl4 gene in SZ95 sebocytes following Acsl4 siRNA-mediated knockout. **(C)** LASER confocal microscopy was utilized to visualize and assess the levels of lipid reactive oxygen species (ROS) in SZ95 sebocytes following Au-Ag-PDA and Au-Ag-PDA@MSCM-mediated PTT treatments. The PTT conditions included a nanoparticle concentration of 40 μg per milliliter, an irradiation time of 8 min. Additionally, the lipid ROS levels were also evaluated in SZ95 sebocytes after knockdown of Acsl4. Scale bar: 10 μm. **(D–F)** Consistent with the treatment conditions affecting lipid ROS levels, biochemical indicators of ferroptosis, such as iron (Fe^2+^), malondialdehyde (MDA), and glutathione (GSH), were detected and measured. **(G)** The quantitative fluorescence analysis conducted using ImageJ software. **(H)** The protein expression level of Acsl4 in SZ95 sebocytes was successfully knocked down using siRNA, consistent with the conditions described above. This knockdown was achieved by introducing Acsl4-specific siRNA into the cells, resulting in a reduction in Acsl4 protein expression. Furthermore, the protein expression level of Acsl4 in SZ95 sebocytes after PTT treatment was detected. **(I)** To analyze the protein expression levels of Acsl4, gray scale analysis was performed using ImageJ software.

We determined whether Au-Ag-PDA@MSCM-mediated PTT induces ferroptosis in SZ95 by modulating Acsl4 expression. To validate our findings, we employed siRNAs to knock out Acsl4 in SZ95 cells (Figure 5A). The protein and transcript levels of Acsl4 in SZ95 cells were subsequently assessed using WB and qRT-PCR (Figures 5B,H).

We evaluated lipid peroxidation levels using C11 BODIPY 581/591 and quantified fluorescence using ImageJ software. Additionally, the expression levels of GSH, MDA, and Fe^2+^ in SZ95 cells were investigated (Figures 5D–F). Following Au-Ag-PDA-mediated PTT, SZ95 cells exhibited increased Fe^2+^ concentrations, elevated MDA levels, and GSH depletion compared with that in normal cells. Additionally, these cells displayed enhanced Lipid Peroxidation fluorescence, as visualized using confocal fluorescence microscopy (Figures 5C,G). In our experiments, both Au-Ag-PDA- and Au-Ag-PDA@MSCM-mediated PTT triggered ferroptosis and upregulation of the Acsl4 protein (Figures 5H,I) in SZ95 cells (*p* < 0.05). However, the effect was more pronounced for Au-Ag-PDA@MSCM. Moreover, when Acsl4 was knocked down in SZ95 cells, PTT-mediated downregulation of Lipid Peroxidation, MDA, and Fe^2+^ was observed, along with reduced Acsl4 protein expression (Figures 5C–I), with no significant GSH depletion.

### 3.6 Ferroptosis occurs during the early stages of Au-Ag-PDA@MSCM-mediated PTT

To validate our findings, we designed animal experiments to negate the confounding effects of the C. acnes control group. This involved the direct application of Au-Ag PDA@MSCM NPs to the sebaceous gland tissues of golden hamster flanks, with tissue samples subsequently collected for WB, HE staining, ORO staining, and Transmission electron microscopy (TEM) immediately after treatment and on days 3 and 5. This setup was crucial for ascertaining the precise timing of ferroptosis and substantiating the direct induction of ferroptosis in sebaceous gland tissues by Au-Ag PDA@MSCM-mediated PTT, along with its role in inhibiting sebum secretion.

**FIGURE 6:**
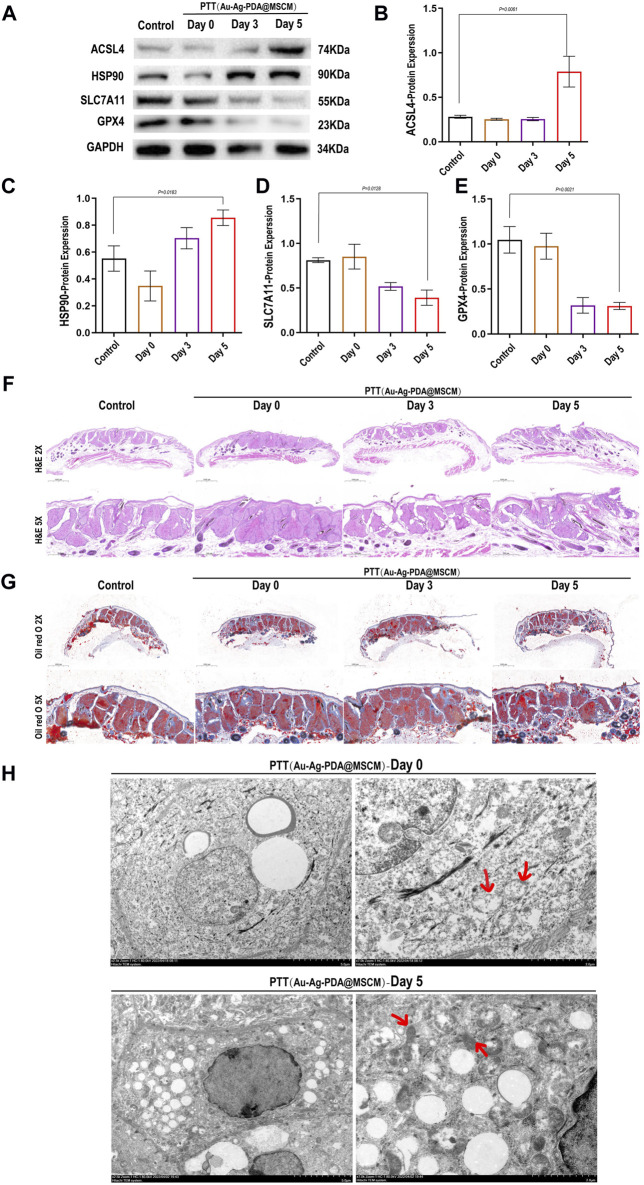
Impact of Au-Ag-PDA@MSCM-mediated PTT on ferroptosis-related protein expression and sebaceous gland ultrastructure. The expression levels of proteins critical to ferroptosis (Acsl4, HSP90, SLC7A11, and GPX4) were determined by western blotting in the Au-Ag-PDA@MSCM-mediated PTT groups **(A–E)**. HE staining **(F)** and Oil Red O **(G)** staining of sebaceous gland cells immediately after 0, 3, and 5 days after photothermal therapy using Au-Ag-PDA@MSCM nanoparticles. Biochemical markers of ferroptosis. Ultrastructural changes of SZ95 sebocytes immediately after and 5 days after PTT with Au-Ag- PDA@MSCM nanoparticles. **(H)** Immediate group and 5 days group. Red arrows indicate mitochondria.

On day 5 post-treatment, we observed a significant elevation in the expression of proteins, notably Acsl4 and HSP90, positively correlated with ferroptosis in tissue samples. Conversely, a marked decrease was observed in proteins negatively correlated with ferroptosis, such as solute carrier family 7 member 11 (SLC7A11) and glutathione peroxidase 4 (GPX4) (Figures 6A–E). On day 5 post-PTT, the size of the sebaceous gland tissues and sebum secretion were reduced (Figures 6F,G). TEM of the tissues revealed that immediately after Au-Ag-PDA@MSCM-mediated PTT, the mitochondria were of normal size with intact membranes, although partial vacuolation was observed (Figure 6H). On day 5, the mitochondria exhibited shrinkage or atrophy, increased membrane density, and loss of cristae. These morphological characteristics are indicative of ferroptotic changes (Figure 6H) (Miyake et al., 2020).

### 3.7 Expression levels of proteins related to the Hippo signaling pathway in sebaceous glands

**FIGURE 7:**
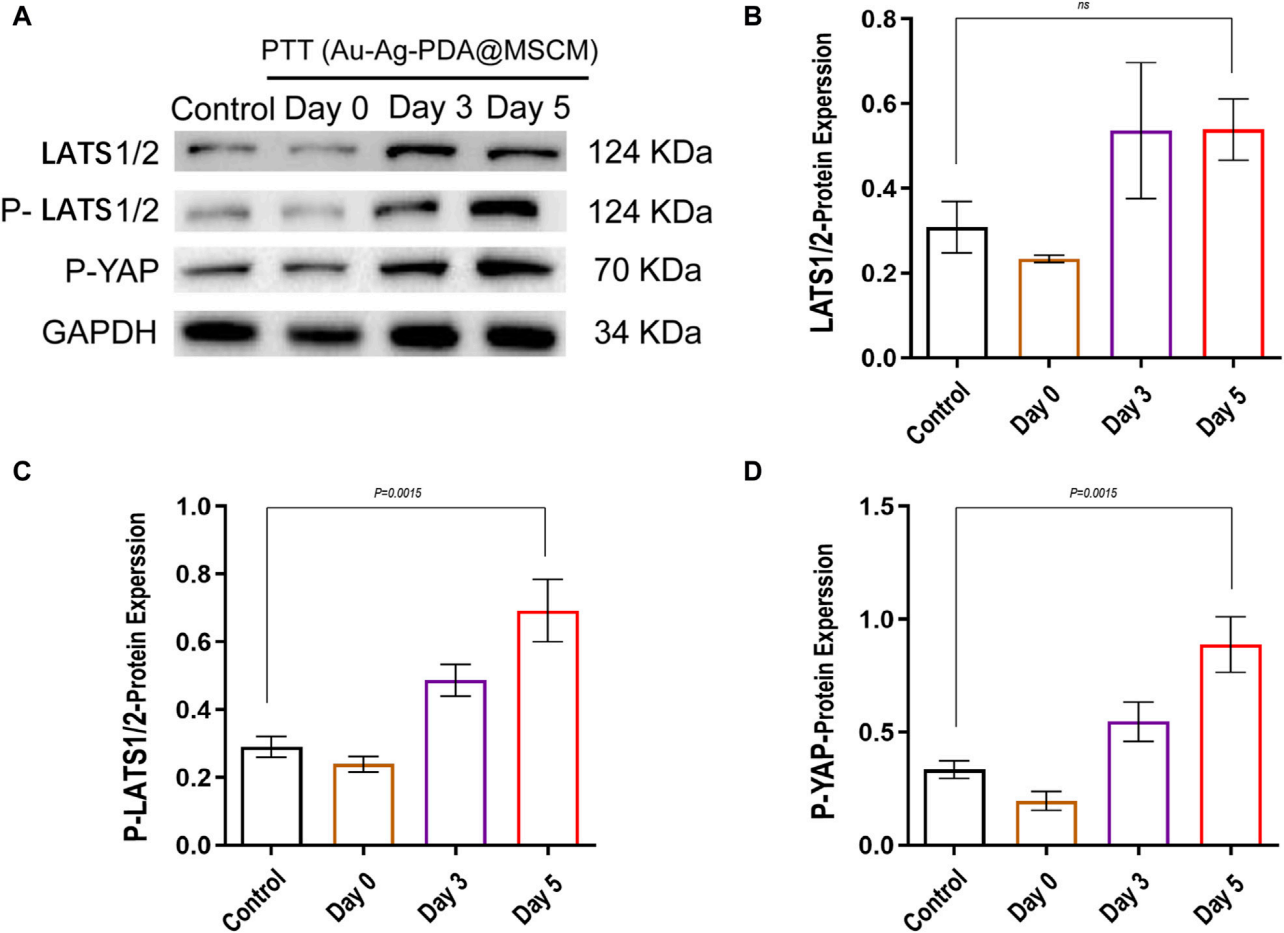
Modulation of Hippo signaling pathway proteins in sebaceous tissue following photothermal therapy mediated by Au-Ag-PDA@MSCM.Sebaceous tissue samples were collected from groups subjected to photothermal therapy mediated by Au-Ag-PDA@MSCM.The levels of critical proteins involved in the Hippo signaling pathway, including LATS1/2, p-LATS1/2, and p-YAP, were determined by western blotting in the groups treated with photothermal therapy mediated by Au-Ag-PDA@MSCM. **(A)** shows the expression changes of these proteins immediately after treatment, at 3 days, and at 5 days. **(B–D)** present the grayscale analysis of the western blotting results using ImageJ.

To clarify the impact of Au-Ag-PDA@MSCM-mediated PTT on the Hippo signaling cascade within sebaceous glandular cells and to validate our proteomic findings, we examined the fluctuating dynamics of Hippo signaling levels in sebaceous gland tissue immediately, as well as on days 3 and 5 following the administration of Au-Ag-PDA@MSCM-mediated PTT. We comprehensively analyzed the phosphorylation dynamics of YAP (p-YAP) and its upstream modulators, LATS1/2, which are the main downstream effectors of the Hippo signaling pathway. The levels of p-YAP and p-LATS1/2 did not change significantly immediately after Au-Ag-PDA@MSCM-mediated PTT, whereas they gradually increased from days 3–5 after Au-Ag-PDA@MSCM-mediated PTT compared with their levels in the blank control group. These protein levels changed most significantly on day 5 (Figures 7A–D); however, we observed no significant change in LATS1/2 levels after this time.

## 4 Discussion

The key pathological features in the development of acne vulgaris include excessive sebum production by the sebaceous glands and sebocyte hyperplasia, leading to the blockage of follicular openings and an inflammatory response. Effectively alleviating the skin damage caused by acne and the oversecretion of sebum has become a pressing issue. Previously, we demonstrated that Au-Ag-PDA@MSCM, a novel low-concentration photosensitizer, significantly reduced sebaceous gland size and sebum secretion. Furthermore, previous studies have demonstrated that low concentrations of Au-Ag-PDA@MSCM NPs are non-toxic in both *in vivo* acne models and *in vitro* SZ95 cells (Tianqi et al., 2020). Additionally, cell membrane coatings provide a stabilizing effect, aiding the uptake of NPs by cells through endocytosis (Gao et al., 2016). However, the mechanism underlying photothermally mediated sebum inhibition is not well understood. Understanding the mechanisms underlying PTT-induced cell death holds significance for clinical applications of PTT. Therefore, we investigated the molecular mechanisms behind sebaceous gland contraction mediated by Au-Ag-PDA@MSCM following 808 nm laser irradiation using both *in vivo* and *in vitro* experiments. Using proteomic approaches, we identified Acsl4 as a key protein that facilitates ferroptosis onset in sebaceous gland cells. Collectively, we present the first detailed elucidation of the precise mechanisms underlying PTT-mediated sebaceous gland cell (SZ95 sebocyte) death and tissue inhibition. These findings provide a theoretical foundation for the application of nanomaterials in the clinical management of acne-like conditions.

While the application of PTT for acne is in its nascent stages, the exact relationship between PTT and sebaceous gland dynamics remains to be elucidated. Owing to the abundance of PUFAs and phospholipids within sebaceous gland tissues, which are key sensitizers for ferroptosis (Zou et al., 2020), these glands exhibit an elevated susceptibility to ferroptosis. In our study, GO enrichment analysis of DEPs revealed that Au-Ag PDA@MSCM-mediated PTT inhibited cellular respiratory processes and oxidative phosphorylation in sebaceous glands. Subcellular localization of these proteins revealed that 30.77% of the downregulated proteins, including cytochrome c oxidase subunit 5A (LOC101843749), which is part of mitochondrial respiratory chain complex IV, were located in the mitochondria (Table 1). Mitochondria are the “powerhouses” of cells that provide ATP through oxidative phosphorylation. They are also crucial in regulated cell death, including apoptosis, pyroptosis, necrosis, and ferroptosis. Gao et al., 2019 demonstrated the critical involvement of mitochondria in cysteine deficiency-induced ferroptosis, demonstrating the vital role of the TCA cycle in cysteine-mediated ferroptosis and highlighting the role of electron transport chain (ETC) in ferroptosis occurrence. Glutamine breakdown and the TCA cycle are important steps in regulating the ferroptosis-associated mitochondrial membrane potential. Consequently, leveraging the insights from the DEP, GO enrichment, and KEGG signaling pathway analyses within our proteomic studies, we postulated that Au-Ag-PDA@MSCM-mediated PTT could induce ferroptosis in sebaceous gland tissues.

To substantiate this hypothesis, we employed SZ95 sebaceous gland cells in our *in vitro* investigations. Using the Calcein AM, EthD-I assay, we confirmed that both Au-Ag-PDA- and Au-Ag-PDA@MSCM-mediated PTT augmented cell death in SZ95 cells. Conversely, treatment with Fer-1 mitigated this increase in cell death. Substantial evidence indicates that IGF-1 stimulates sebum production in SZ95 sebaceous gland cells, with a concomitant positive correlation between IGF-1 levels and clinical acne severity (Im et al., 2012). Consequently, we administered Au-Ag-PDA- and Au-Ag-PDA@MSCM-mediated PTT following the induction of sebum secretion in SZ95 cells with IGF-1 and observed that PTT effectively diminished sebum secretion in these cells. The results demonstrated that PTT effectively diminished sebum secretion in these cells, and the irregular morphology suggested their imminent demise. Importantly, the application of a ferroptosis inhibitor abrogated these effects.

To definitively confirm the pivotal molecular targets, we validated Acsl4 (Table 1), which was identified as upregulated in our enriched proteomic analysis. Although the upregulation of Acsl4 did not reach statistical significance in our proteomic results, we chose to further validate Acsl4 based on our bioinformatic analysis and the absence of other programmed cell death-related proteins among the 3211 differentially expressed proteins. Kagan et al. reported that Acsl4 plays a crucial role in modulating the lipidomic profile of cells, influencing their susceptibility to ferroptosis, and determining the balance between sensitivity and resistance to this form of cell death (Doll et al., 2017). Miao et al. reported that in the temperature range of 37°C–65°C, overexpression of HSP90 is vital to maintaining the stability of Acsl4 expression within glioma cells (Miao et al., 2022). In this study, siRNA transfection was used to inhibit Acsl4 expression. Our data revealed that subsequent administration of both Au-Ag-PDA- and Au-Ag-PDA@MSCM-mediated PTT heightened lipid peroxidation markers (lipid ROS, MDA), augmented sub-Fe ion levels, and depleted GSH. Notably, silencing Acsl4 in SZ95 cells prior to PTT exposure mitigated ferroptosis. The results obtained from the *in vitro* experiments further confirmed that Au-Ag-PDA mediated PTT-induced ferroptosis in sebaceous gland cells (SZ95 sebocytes) by upregulating Acsl4. Although the *p*-values did not achieve statistical significance, nanoparticles encapsulated with MSCM exhibited a substantial enhancement in mediating ferroptosis.It is postulated that the principal mechanism involves thermogenic mediation, with MSCM primarily facilitating the delivery process. Consequently, although the transdermal properties of MSCM were not significantly apparent *in vitro*, our preliminary *in vivo* studies demonstrated that MSCM-encapsulated nanoparticles displayed pronounced targeting and enhanced transdermal capabilities within the skin (Tianqi et al., 2020).

The mechanistic foundations of ferroptosis are well-established and include two primary pathways, notably the System Xc-/GpX4 pathway. In this pathway, cysteine is transported into the cell via SLC7A11 and SLC3A2 dimers (System Xc-) embedded in the cellular membrane, where it is subsequently oxidized to cysteine. Cysteine is enzymatically converted into GSH by the concerted action of gamma-glutamylcysteine ligase (GCL) and glutathione synthetase (GSS). GPX4 uses GSH to convert the peroxyl bond (l-OOH) of lipid peroxidation into a less reactive hydroxyl group (l-OH), effectively neutralizing its peroxidative capacity. This biochemical cascade acts as a defense against ferroptosis, and SLC7A11 plays a pivotal role in this process (Chen et al., 2021). The lipid metabolism pathway is an alternative canonical pathway implicated in ferroptosis. PUFAs are conjugated to phosphatidylethanolamine (PE) via the enzymatic action of Acsl4 to yield PUFA phospholipids (PUFA-PE). These PUFA-PE molecules are particularly vulnerable to oxidation via lipoxygenase-mediated free radicals, thereby catalyzing the onset of ferroptosis (Liu et al., 2021). Furthermore, there is a notable upsurge in the proportion of HSP90 within the total cellular proteome in response to cellular stress, escalating from a baseline of 1%–2% to 4%–6%. Concurrently, HSP90 stabilizes Acsl4 expression, thereby facilitating ferroptosis in glioma cells (Taipale et al., 2010; Finka and Goloubinoff, 2013; Miao et al., 2022).

To ascertain the onset of ferroptosis following PTT, Au-Ag-PDA@MSCM was topically administered to the bilateral flank sebaceous glands of hamsters. We procured various tissue samples, including blank controls, immediate post-PTT, and tissues from days 3 and 5 post-treatment. WB was performed to identify crucial ferroptosis-associated proteins. Notably, the findings indicated pronounced upregulation of Acsl4 and Hsp90, along with significant downregulation of Slc7a11 and Gpx4 in the sebaceous gland tissues by day 5 post-PTT. Our assessment of ferroptosis biomarkers, coupled with detailed tissue TEM and both HE and ORO staining, substantiated the induction of ferroptosis in sebaceous gland tissues by Au-Ag PDA@MSCM-mediated PTT and its subsequent inhibition of sebum secretion. This evidence substantiates the initiation of ferroptosis in these tissues on day 5, mediated by Au-Ag-PDA@MSCM. Subsequently, we investigated the signaling pathways potentially implicated in PTT-mediated ferroptosis within sebaceous glands.

The Hippo pathway is a conserved signaling pathway that regulates organ function by regulating cell proliferation and apoptosis (Pan, 2010; Yu et al., 2015). Recent studies have focused on the regulatory role of the Hippo pathway in ferroptosis. Notably, heat stress has emerged as a significant upstream signal capable of activating YAP/TAZ through HSP90, thereby inducing the heat shock transcriptome (Luo et al., 2020). YAP is a key regulator of the Hippo signaling pathway. YAP/TAZ target genes regulate ferroptosis in multiple ways, including by regulating the expression of Acsl4, SLC7A11, and transferrin receptor (TFRC), as well as ROS production (Magesh and Cai, 2022; Zhang et al., 2022). WB analysis indicated that Au-Ag-PDA@MSCM-mediated PTT facilitated the phosphorylation of Lats1/2 and Yap on day 5 post-treatment, signifying a pivotal modulation of cellular signaling pathways. Furthermore, HE and ORO staining revealed a noticeable reduction in the size of sebaceous gland tissues and a decrease in sebum production on day 5 following PTT. TEM of tissue samples from the same day revealed mitochondrial crista attenuation and loss. This *in vivo* study unequivocally established that Au-Ag-PDA@MSCM-mediated PTT triggered ferroptosis in sebaceous gland tissues, indicating this process initiates on day 5 post-PTT. Moreover, our preliminary findings confirmed that changes in the Hippo signaling pathway occurred on day 5 after PTT.

The clinical application of gold nanoparticle-mediated PTT in acne treatment has been documented, affirming its safety profile (Suh et al., 2021; Seo et al., 2022). However, the mechanisms underlying the use of PTT in acne therapy remain unelucidated. Our initial findings demonstrated the safety and efficacy of Au-Ag-PDA@MSCMs in reducing sebum production (Tianqi et al., 2020). This study utilized proteomic analysis, bolstered by Western Blotting (WB) and small interfering RNA (siRNA) experiments, Preliminary evidence suggests that Au-Ag-PDA@MSCM-mediated PTT unequivocally induces ferroptosis in SZ95 sebaceous gland cells through the upregulation of Acsl4. Furthermore, meticulous *in vitro* experiments substantiated the occurrence of mitochondrial damage in sebaceous gland tissue precisely on the 5th day post-PTT, providing irrefutable evidence for the initiation of ferroptosis. Of note, preliminary observations suggest that the photothermal therapy (PTT)-induced ferroptotic process may involve the inhibition of the Hippo signaling pathway and the System Xc-/GpX4 system. Additionally, utilizing MSCM as a drug delivery system can more effectively induce ferroptosis in SZ95 cells, thus inhibiting sebum secretion.Consequently, future research should delve into how the Hippo signaling pathway intricately regulates ferroptosis in sebaceous gland tissues under PTT conditions.Furthermore, given MSCM’s ability to enhance transdermal properties, employing it to encapsulate ferroptosis inducers and Au-Ag-PDA may yield a more effective method for suppressing sebum secretion and treating acne.

## 5 Conclusion

In summary, our findings demonstrate that Au-Ag-PDA@MSCM-mediated PTT, by modulating Acsl4, effectively induces ferroptosis, thereby inhibiting sebum secretion in sebaceous gland tissues and cells. MSCM, as a drug delivery system, significantly enhances this mechanism.Preliminary *in vitro* studies have confirmed the initiation of ferroptosis in sebaceous gland tissues on the fifth day post-PTT, highlighting the temporal dynamics of this phenomenon.Additionally, we have identified a potential regulatory mechanism involving Acsl4 and the Hippo pathway, which suggests a viable route to augmenting PTT-induced ferroptosis mediated by Au-Ag-PDA@MSCM in sebaceous glands.Overall, our findings not only provide robust theoretical support but also pave the way for a paradigm shift in acne treatment by integrating nanomaterials into PTT.

## Data Availability

The datasets presented in this study can be found in online repositories. The names of the repository/repositories and accession number(s) can be found in the article/Supplementary Material.

## Abbreviations

Acsl4, Acyl-CoA synthetase long-chain family member 4; ALA-PDT, aminolevulinic acid-photodynamic therapy; BCA, bicinchoninic acid; BP, biological process; CC, cellular components; DEP, differentially enriched protein; DMEM, Dulbecco’s modified Eagle’s medium; GSH, glutathione; HAADF, high-angle annular dark-field imaging; LC-MS, liquid chromatography-mass spectrometry; MDA, malondialdehyde; MSCM, mesenchymal stem cell membrane; NIR, near-infrared; NP, nanoparticle; MF, molecular function; PASEF, parallel cumulative serial fragmentation; PDA, polydopamine; PE, phosphatidylethanolamine; PTT, photothermal therapy; PUFA, polyunsaturated fatty acid; qRT-PCR, quantitative reverse transcription PCR; SDS, sodium dodecyl sulfate; STEM, scanning transmission electron microscopy.
