# Supplementary material for: Unveiling the mechanism of photothermal therapy in acne man-agement: targeting sebaceous gland ferroptosis via umbilical cord mesenchymal stem cell membrane-encapsulated Au-Ag-PDA
Source: Front Bioeng Biotechnol. 2024 Jun 10;12:1426477. doi: 10.3389/fbioe.2024.1426477 (PMC11194360; doi:10.3389/fbioe.2024.1426477)
Supplement: Supplementary file 1 [file DataSheet1.docx]

**SUPPLEMENTARY INFORMATION**


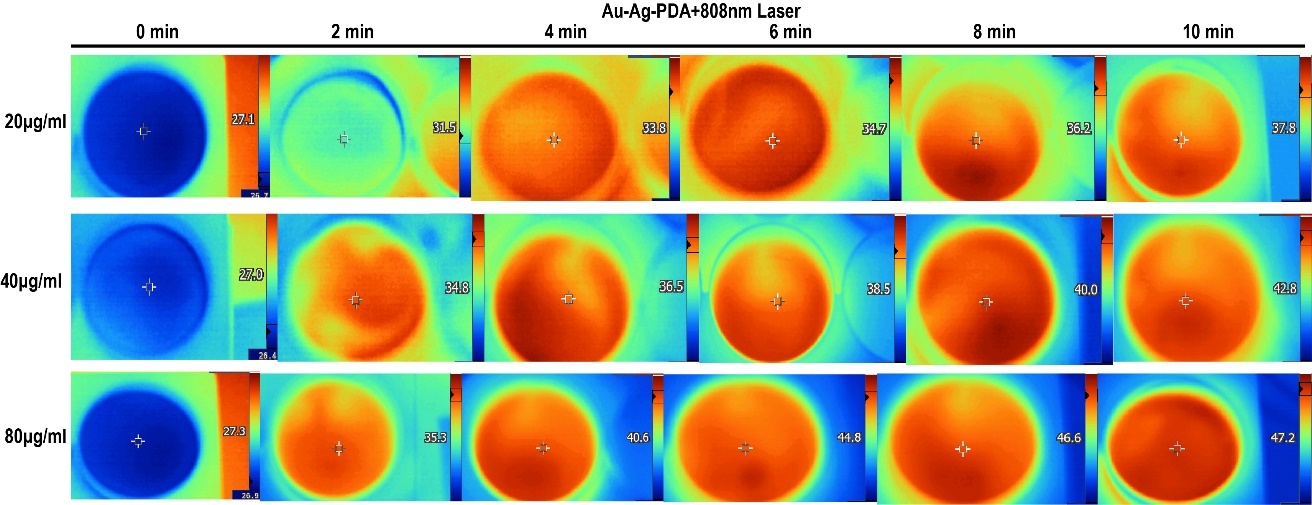


**FIGURE S1** : **Investigation of Temperature Changes in Aqueous Nanoparticle Solutions under 808nm LASER Irradiation.** Aqueous solutions containing nanoparticles at different concentrations (20, 40, 80) were prepared. The irradiation times tested were 0, 2, 4, 6, 8, and 10 min, representing different durations of exposure to the 808nm LASER. By using an infrared imager, we were able to visualize and quantify the temperature changes in the aqueous nanoparticle solutions under different experimental conditions.


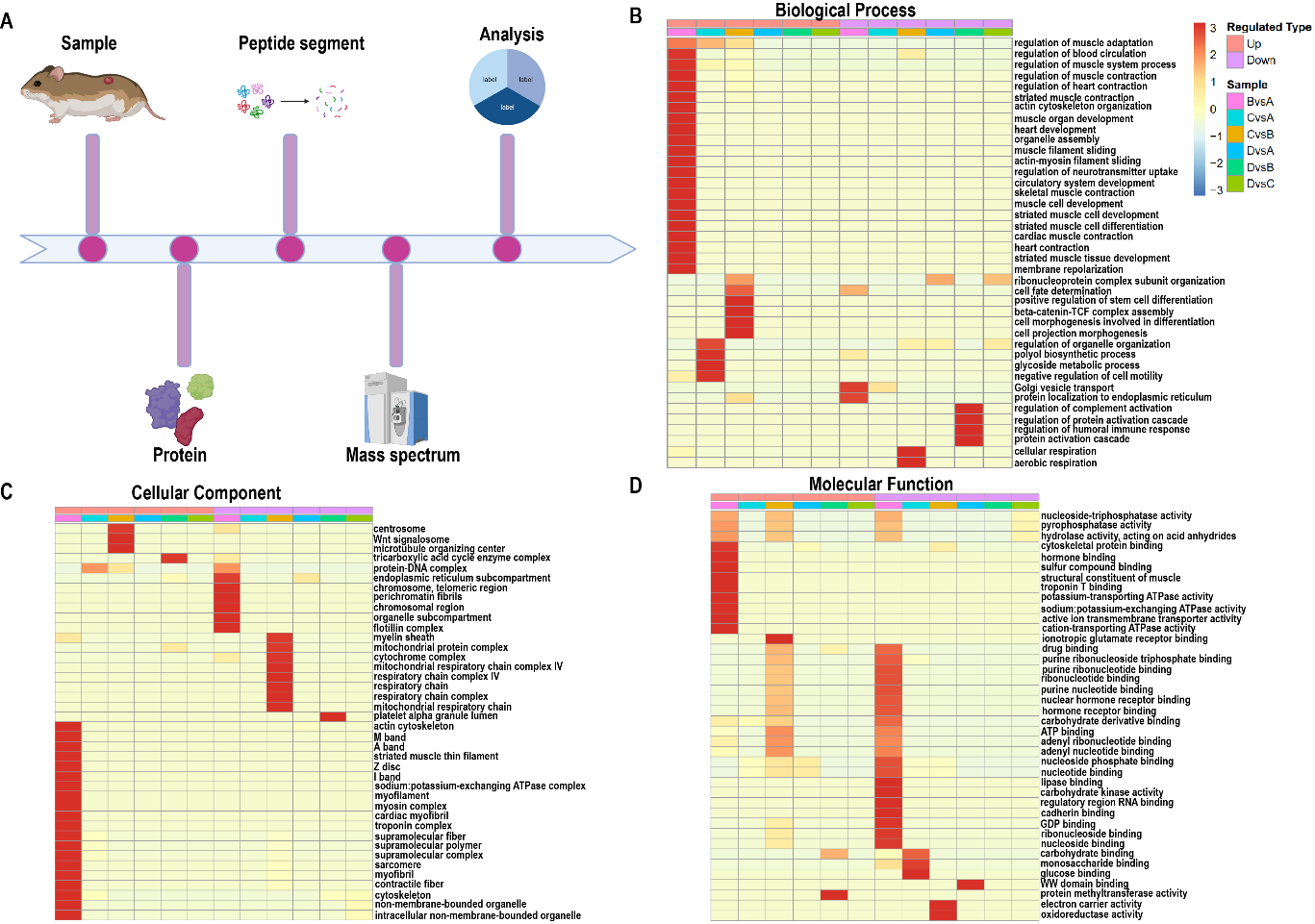


**FIGURE S2:** **Comprehensive Analysis of Proteomic Changes and Gene Ontology Enrichment in Different Experimental Groups: Insights from Biological Process, Cellular Component, and Molecular Function Perspectives.**(A) Proteomics experimental workflow Heat map of the cluster analysis based on the Gene Ontology enrichment classifications of (B) Biological Process (C) cellular component and (D) molecular function. Groups described in Section 2.2 (A: Control, B: C*. acnes*, C: *C. acnes* + Au-Ag-PDA@MSCM, and D: *C. acnes* + Au-Ag-PDA). The horizontal direction indicates the grouping and the vertical direction indicates the described functions. Red color indicates a high degree of enrichment.


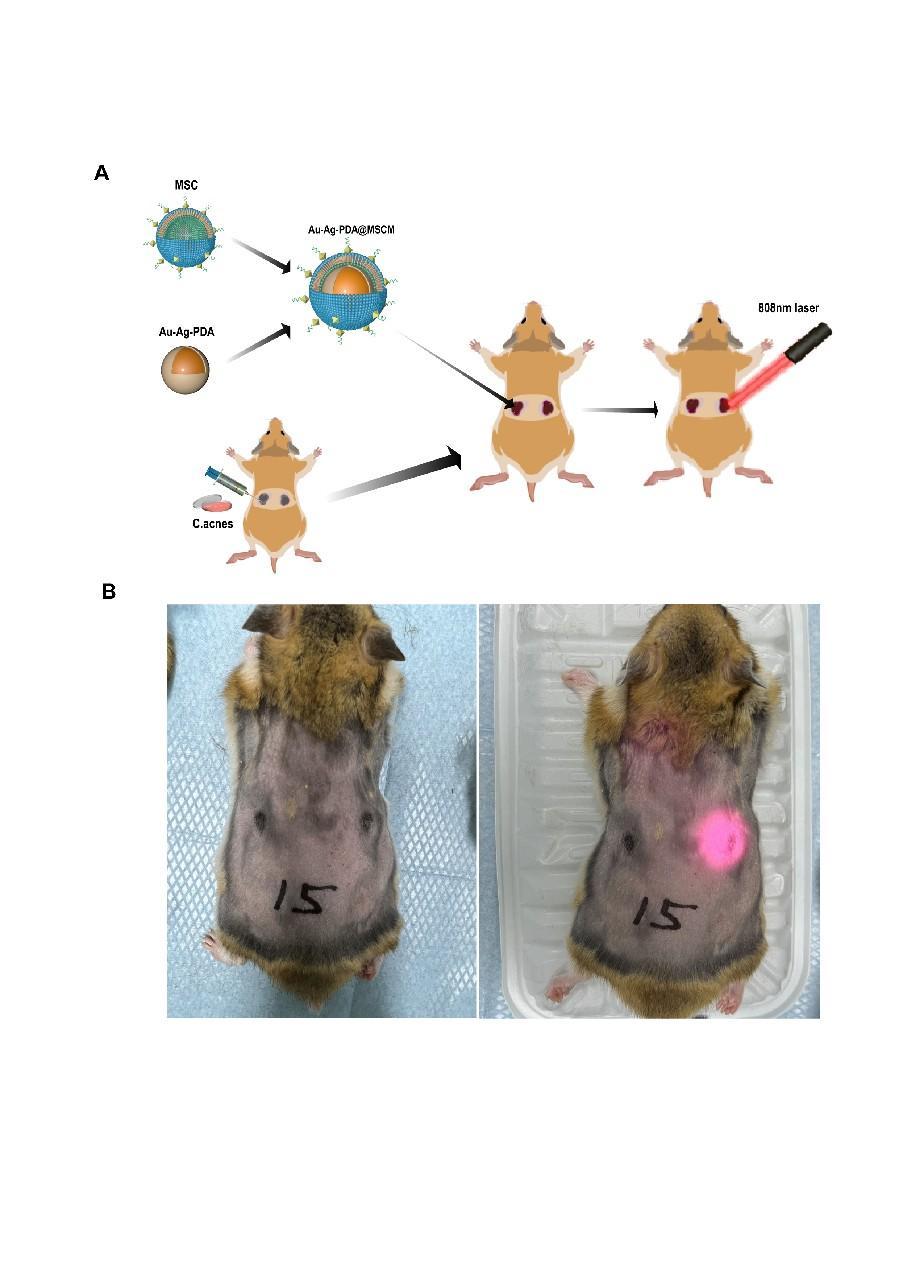


**FIGURE S3:** **Exploring the In Vivo Photothermal Therapy (PTT) Process Using Au-Ag-PDA@MSCM and Au-Ag-PDA Nanoparticles.**In vivo experimental Au-Ag PDA@MSCM or Au-Ag PDA-mediated photothermal therapy (PTT) process.The concentration of Au-Ag-PDAMSCM NPs was 30 μL, 20 μg/mL and the 808 nm laser parameter was 0.5 W/cm2 for 3 min irradiation


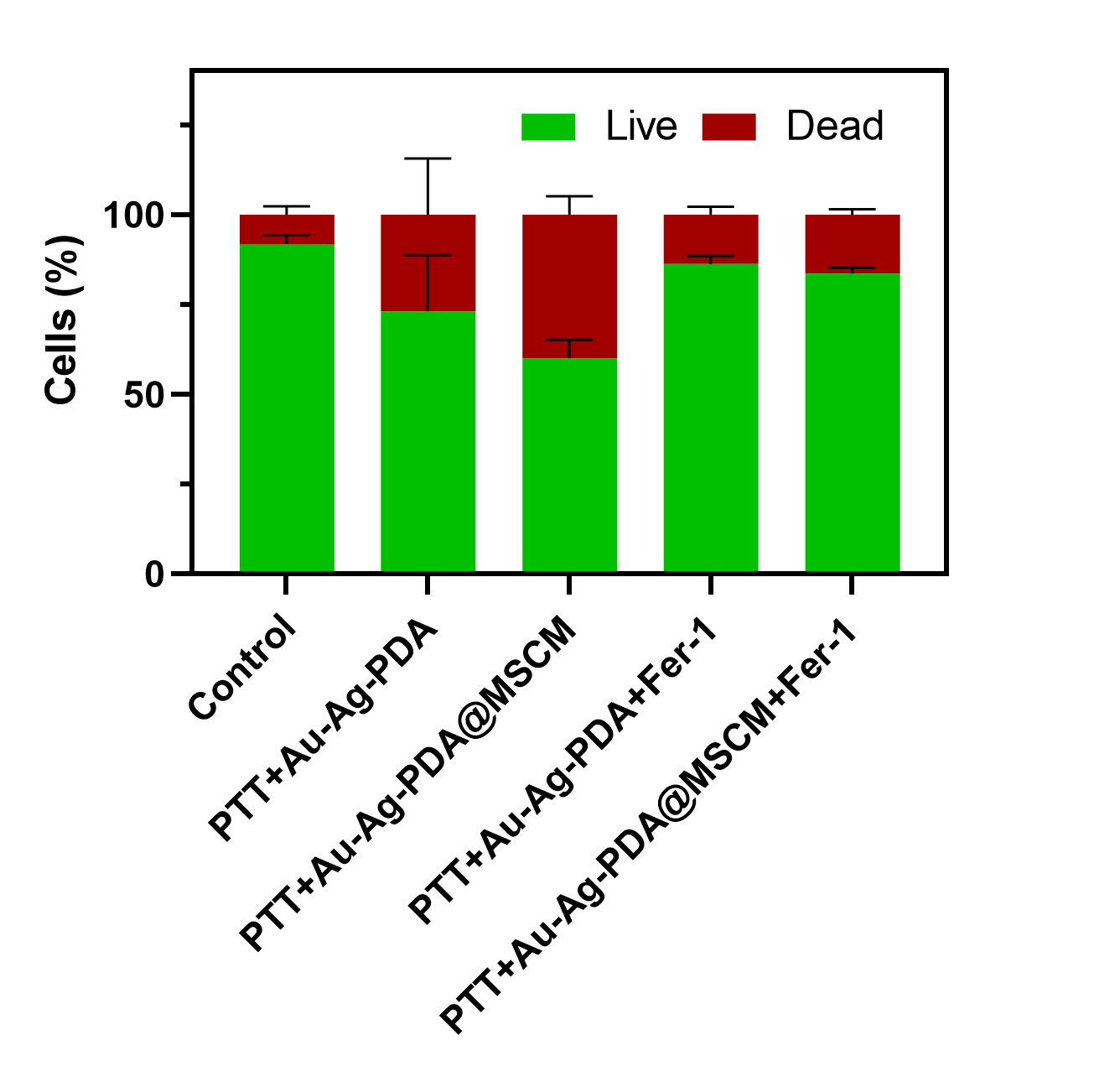


**FIGURE S4:** **Percentage of Live and Dead Cells.**In the statistical analysis using ImageJ, green represents live cells, and red represents dead cells. This chart shows the percentage of live and dead cells in each group.
